# New findings in diabetic kidney disease: correlation and enhanced combined diagnostic value of sclerostin and 25(OH)VD

**DOI:** 10.3389/fendo.2026.1817017

**Published:** 2026-04-13

**Authors:** Chunfan Niu, Jianhong Yin, Mina Li, Qianqian Wu, Ming Liu, Li Sun, Jing Yang, Linxin Xu

**Affiliations:** 1 First Clinical Medical College, Shanxi Medical University, Taiyuan, Shanxi, China; 2 Department of Endocrinology, First Hospital of Shanxi Medical University, Taiyuan, Shanxi, China; 3 Shanxi Innovation Center for Integrated Management of Hypertension, Hyperlipidemia and Hyperglycemia Correlated with Cardiovascular and Cerebrovascular Diseases, Taiyuan, Shanxi, China; 4 Clinical Research Center for Metabolic Diseases of Shanxi Medical University, Taiyuan, Shanxi, China

**Keywords:** 25(OH)VD, sclerostin, type 2 diabetes mellitus, diabetic kidney disease, DKD

## Abstract

**Objective:**

Diabetic kidney disease (DKD) is a major microvascular complication of type 2 diabetes mellitus (T2DM), and its early identification is crucial. As a novel endocrine marker, the relationship between sclerostin and DKD, as well as its combined diagnostic value with 25-hydroxyvitamin D (25(OH)VD), remains unclear. This study aims to investigate circulating sclerostin levels in patients with DKD and its combined diagnostic value with 25(OH)VD, providing evidence for early clinical diagnosis.

**Methods:**

A total of 308 patients with T2DM were enrolled, including 113 with DKD (DKD group) and 195 without DKD (T2DM group). The DKD group was subdivided into microalbuminuria and macroalbuminuria groups based on UACR. General information and clinical indicators were collected for all patients. Concurrently, blood samples were collected to measure serum sclerostin levels using the ELISA method. Statistical analysis evaluated sclerostin expression differences across groups and its correlations with other indicators. Binary logistic regression analyzed the independent associations of sclerostin and 25(OH)VD with DKD. Receiver operating characteristic (ROC) curves were plotted to assess the predictive efficacy of serum sclerostin and 25(OH)VD levels for T2DM with albuminuria.

**Results:**

Compared to the T2DM group, patients in the DKD group exhibited decreased serum sclerostin and 25(OH)VD levels (*P<* 0.05). Further subgroup analysis of DKD revealed that serum sclerostin levels were significantly lower in both the microalbuminuria and macroalbuminuria groups compared to the normal albuminuria group (*P<* 0.05). Serum 25(OH)VD in the massive proteinuria group was significantly lower than in both the normal proteinuria and microalbuminuria groups (*P<* 0.05). Correlation analysis showed a significant negative correlation between sclerostin and UACR (r = -0.197, *P<* 0.001) and a significant positive correlation with 25(OH)VD (r = 0.167, *P* = 0.003). Binary logistic regression analysis demonstrated that serum sclerostin and 25(OH)VD remained independent predictors of DKD even after adjusting for variables. ROC curve analysis showed that the AUC for predicting DKD using serum sclerostin and 25(OH)VD was 0.73, with a sensitivity of 61.1% and specificity of 75%.

**Conclusion:**

This study confirms that serum levels of sclerostin and 25(OH)VD are significantly reduced in patients with DKD, and both are independent protective factors for DKD. Their combined assessment demonstrates good predictive value for the early identification of DKD, providing clinical insights into the interaction between bone metabolism and renal pathology.

## Introduction

Diabetes mellitus is a chronic metabolic disorder characterized by persistent hyperglycemia resulting from insufficient insulin secretion or impaired insulin action (1). According to the latest report from the International Diabetes Federation, the global prevalence of diabetes continues to rise, with an estimated 853 million people affected by 2050 (2). Type 2 diabetes mellitus (T2DM) is the most common form of diabetes, accounting for over 90% of global cases (3). The long-lasting elevation of blood glucose can trigger multiple complications, among which diabetic kidney disease (DKD) represents a common and severe microvascular complication of T2DM, affecting approximately 30-40% of individuals with diabetes (4). DKD involves a complex pathogenesis, primarily characterized by persistent metabolic dysfunction, inflammation, oxidative stress, and fibrotic processes (5–9). Clinically, it manifests as persistent proteinuria and/or progressive decline in glomerular filtration rate (GFR), serving as a primary cause of chronic kidney disease (CKD) and end-stage kidneys disease (ESKD) (10). ESRD not only impairs renal function but also increases the risk of cardiovascular disease and mortality, significantly compromising patient health (11, 12). In clinical practice, the urine albumin-to-creatinine ratio (UACR) is a commonly used indicator for screening DKD. However, UACR is influenced by multiple factors and lacks specificity in the early stages of DKD. Therefore, identifying serum biomarkers for early DKD diagnosis and implementing timely interventions are crucial for delaying renal function deterioration.

Sclerostin is a glycoprotein primarily secreted by osteocytes, acting as a natural inhibitor of the Wnt/β-catenin signaling pathway. While its classic role is regulating bone metabolism, recent studies reveal this protein is also expressed in cardiovascular tissues, kidneys, liver, brain, and adipocytes, participating in the pathogenesis of diseases in these organs (13–15). Sclerostin is implicated in various conditions including type 2 diabetes, cardiovascular disease, and liver cirrhosis. Research indicates that Wnt/β-catenin signaling pathway plays a crucial role in renal pathophysiology (16, 17). Animal studies have demonstrated that deletion of the sclerostin gene (Sost) exacerbates renal interstitial fibrosis in mice, whereas sclerostin treatment can ameliorate renal fibrosis (18, 19). However, the pattern of circulating sclerostin level changes in DKD and its clinical significance remain unclear.

25-hydroxyvitamin D (25(OH)VD) is the primary storage form of vitamin D in the body and serves as the gold standard for assessing vitamin D nutritional status. While its classic role involves regulating calcium-phosphorus metabolism and maintaining bone health, recent studies reveal its involvement in glucose regulation, immune modulation, vascular homeostasis, and inflammatory processes (20, 21). Studies indicate that 25(OH)VD deficiency is prevalent in T2DM, and low 25(OH)VD levels are significantly associated with renal function decline in T2DM patients (22, 23). However, the clinical application of either circulating sclerostin alone or its combination with 25(OH)VD in DKD remains unclear. This study aims to investigate the relationship between circulating sclerostin levels and DKD, as well as the diagnostic value of combined 25(OH)VD assessment, to provide evidence for early prevention and management of DKD.

## Materials and methods

### Research subjects

This study ultimately enrolled 308 patients with T2DM aged 18–70 years who visited the Endocrinology Department of the First Hospital of Shanxi Medical University between March 2025 and October 2025. Among them, 113 patients were diagnosed with DKD, and 195 patients were without DKD. The diagnostic criteria for T2DM were in accordance with the 2024 American Diabetes Association guidelines (1): hemoglobin A1c (HbA1c) ≥ 6.5%; and/or fasting plasma glucose (FPG) ≥ 7.0 mmol/L; and/or 2-hour plasma glucose ≥ 11.1 mmol/L during an oral glucose tolerance test (OGTT); and/or random plasma glucose ≥ 11.1 mmol/L accompanied by diabetes-related symptoms and signs. The diagnostic criteria for DKD were: established diagnosis of T2DM with exclusion of other chronic kidney diseases, and UACR ≥ 30 mg/g and/or eGFR < 60 mL/min/1.73 m² persisting for more than 3 months. Exclusion criteria: (1) Type 1 diabetes or other special types of diabetes; (2) Patients with diabetic ketoacidosis or hyperosmolar diabetic coma; (3) Patients with acute or severe infection; (4) Kidney diseases not caused by diabetes; (5) Patients with malignant tumors; (6) Patients with communication barriers due to comorbid psychiatric disorders; (7) Inability to cooperate with this study. Patients were primarily divided into the T2DM group (UACR< 30 mg/g) and the DKD group (UACR ≥ 30 mg/g). To further observe various indicators and the progression of proteinuria, patients were subdivided based on UACR values into: normal albuminuria group (UACR< 30 mg/g), microalbuminuria group (UACR 30–300 mg/g), and macroalbuminuria group (UACR > 300 mg/g). This study was approved by the Ethics Committee of the First Hospital of Shanxi Medical University (approval number: KYLL-2025-001), and all patients provided written informed consent.

### Research methods

#### Data collection

On the day of the patient’s visit, information including age, gender, diabetes duration, and medical history was recorded. Height, weight, waist circumference, and blood pressure were measured. Body mass index (BMI) was calculated as weight in kilograms divided by the square of height in meters (kg/m²). All patients fasted for 8–10 hours, and venous blood samples were collected between 6:00 AM and 8:00 AM for the measurement of fasting plasma glucose (FPG), total cholesterol (TC), triglycerides (TG), high-density lipoprotein cholesterol (HDL-C), low-density lipoprotein cholesterol (LDL-C), uric acid (UA), and serum creatinine (Scr) using a Beckman Automatic Biochemical Analyzer (USA, BK-200). Glycated hemoglobin A1c (HbA1c) levels were determined by high-performance liquid chromatography (Roche 501, Switzerland). Serum 25(OH)VD concentrations were measured using chemiluminescence assay kits provided by Roche Diagnostic Products (Shanghai) Co., Ltd., with within-batch and between-batch coefficients of variation (CV) of<8% and<10%, respectively. Urine samples were also collected to measure urinary microalbumin (UmALB) and creatinine (Ucr) levels, and the urinary albumin-to-creatinine ratio (UACR) was calculated (mg/g). The estimated glomerular filtration rate (eGFR) was calculated using the CKD-EPI formula:


$$eGFR=a\times \text{min}{\left(\frac{Scr}{k},\;1\right)}^{\alpha }\times \text{max}{\left(\frac{Scr}{k},\;1\right)}^{-1.209}\times 0.993^{Age}$$

where for males a=141, k=0.9, α=-0.411, and for females a=144, k=0.7, α=-0.329.

#### Measurement of serum sclerostin

All patients fasted for 8–10 hours prior to peripheral venous blood collection. The samples were centrifuged at 3000 rpm for 15 minutes (centrifugal radius: 13.5 cm) to obtain the supernatant, which was then stored frozen at -80 °C. Serum sclerostin levels were measured using an enzyme-linked immunosorbent assay (ELISA) kit (Hepeng (Shanghai) Biotechnology Co., Ltd.). The intra-assay coefficient of variation (CV) was<4.7%, and the inter-assay CV was<9.0%. All measurements were performed in duplicate, and all procedures were strictly carried out in accordance with the manufacturer’s instructions for both the kit and the instruments.

#### Statistical methods

Statistical analysis was performed using SPSS (version 27.0). Continuous variables following a normal distribution are presented as mean ± standard deviation (x̄ ± s), while those with a non-normal distribution are expressed as median with interquartile range (M (Q1, Q3)). Categorical variables are presented as number (percentage). For two-group comparisons, the independent samples t-test was used for normally distributed data and the Mann-Whitney U test for non-normally distributed data. For comparisons among multiple groups, one-way analysis of variance (ANOVA) was used for normally distributed data, with the LSD test employed for *post hoc* pairwise comparisons. The Kruskal-Wallis test was used for non-normally distributed data across multiple groups, followed by *post hoc* pairwise comparisons. Spearman’s rank correlation analysis was used to assess the correlations between UACR and sclerostin as well as other variables. Binary logistic regression analysis was performed to evaluate the independent association of serum sclerostin and 25(OH)VD levels with diabetic kidney disease. The diagnostic value of these markers for diabetic kidney disease was determined by plotting receiver operating characteristic (ROC) curves.

## Results

### Demographics and baseline clinical characteristics of the study population

**Figure 1:**
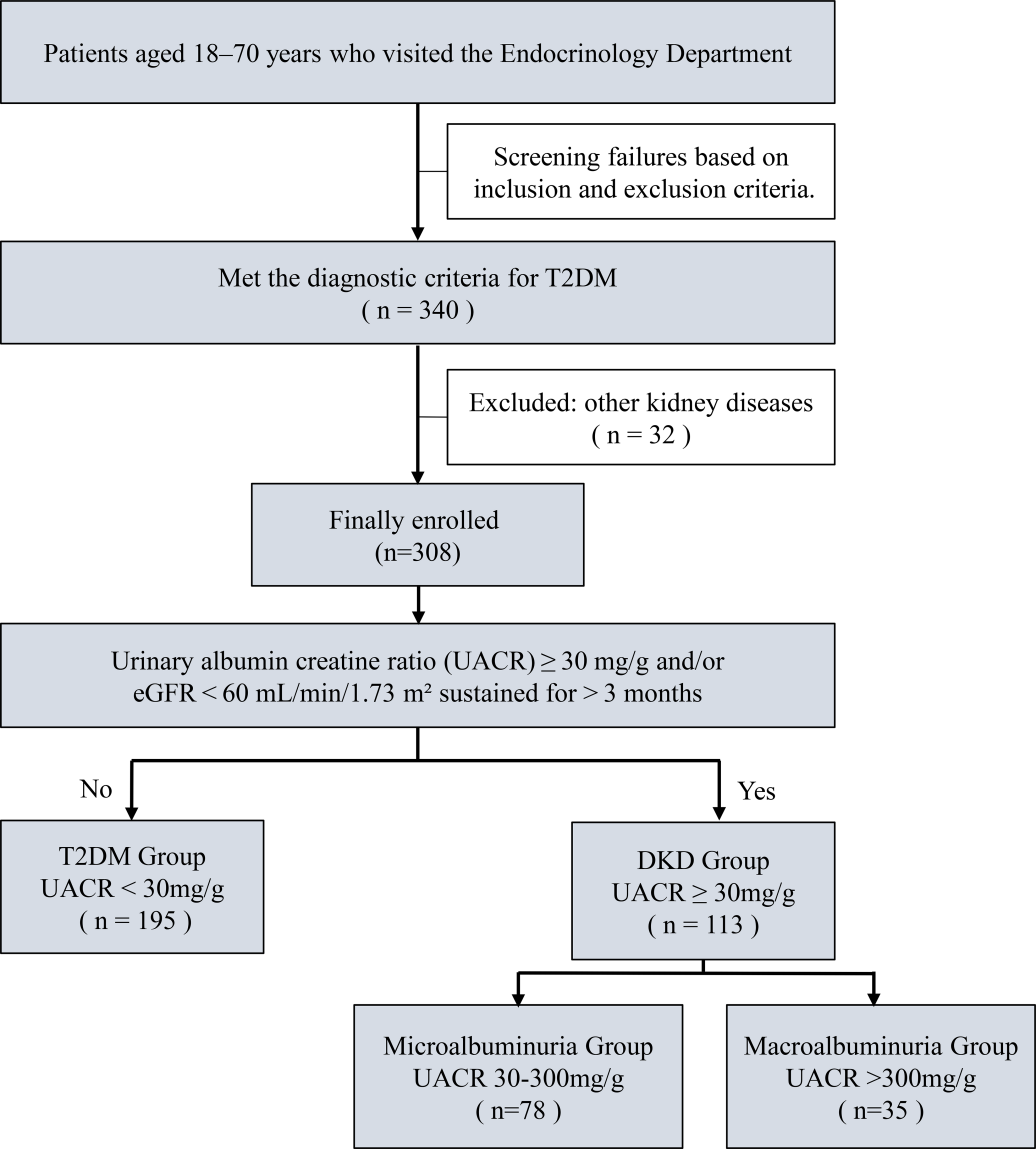
Flow chart of patient enrollment and grouping.

308 patients with T2DM ultimately met the inclusion criteria and participated in this study, comprising 188 males (61%) and 120 females (39%), with a mean age of 57 years. Among them, 195 patients (63.3%) were in T2DM group (normal proteinuria), and 113 patients (36.7%) were in DKD group. The DKD group was further subdivided into microalbuminuria group (78 patients, 25.3%) and macroalbuminuria group (35 patients, 11.4%) (**Figure 1**).

The baseline clinical characteristics of the study population are shown in **Table 1**. There were no significant differences in age, gender, BMI, TC, HDL-C, and LDL-C among the groups. Comparisons between the T2DM and DKD groups (*P*_a_) revealed that DKD patients had longer diabetes duration, higher blood pressure, HbA1c, TG, UA, and Scr, and significantly reduced eGFR (*P<* 0.05). Further divided by Albuminuria severity, overall comparisons (*P*_b_) among the three groups revealed significant intergroup differences in the aforementioned indicators. *Post-hoc* analyses indicated that although some indicators showed no significant statistical differences within subgroups, their values exhibited a gradient change with increasing severity.

**Table 1:** Comparison of baseline clinical characteristics among T2DM patients grouped by Albuminuria status and severity.

Variable	T2DM Group	DKD Group		*P*_a_	*P*_b_
	Normal albuminuria (UACR<30mg/g)	Microalbuminuria (UACR 30-300mg/g)	Macroalbuminuria (UACR>300mg/g)		
Age (years)	59 (51,65)	61 (52,67.5)	56 (47.75,63.25)	0.253	0.14
Gender (M/F)	116/79	47/31	25/10	0.463	0.405
Duration (years)	10 (4,16)	10 (6.5,20)^*^	14.5 (7.5,20)^*^	0.006	0.02
BMI (kg/m^2^)	24.22 (22.49,26.42)	25.35 (22.95,27.24)	25.71 (21.45,27.69)	0.113	0.183
SBP (mmHg)	128 (117,140)	139.63 ± 20.29^***^	147.35 ± 22.27^***^	<0.001	<0.001
DBP (mmHg)	81.5 (73.25,89.75)	84 (74.5,94)	90 (77.5,100.5)^**^	0.01	0.008
FPG (mmol/L)	7.21 (5.73,9.39)	8.83 (6.63,11.45)^*^	7.19 (5.75,9.44)	0.067	0.037
HbA1c (%)	8.37 (7.15,9.74)	9.32 (8.28,10.58)^**^	9.31 (7.24,11.08)	0.003	0.012
TC (mmol/L)	4.36 (3.44,5.05)	4.23 (3.53,5.02)	4.85 (3.4,6.55)	0.567	0.135
TG (mmol/L)	1.50 (1.02,2.38)	1.75 (1.36,3.07)^*^	1.75 (0.92,2.87)	0.023	0.049
HDL-C (mmol/L)	1.08 (0.92,1.25)	1.04 (0.93,1.2)	1.07 (0.95,1.37)	0627	0.395
LDL-C (mmol/L)	2.73 (2.12,3.22)	2.66 (2.12,3.16)	3.06 (2.15,4.2)	0.49	0.221
UA (mmol/L)	301 (238.5,367.25)	317 (252.5,389)	373 (304.25,450.75)^***,††^	0.006	<0.001
Scr (mg/dL)	0.67 (0.55,0.75)	0.71 (0.59,0.84)^*^	1.03 (0.68,1.39)^***,†††^	<0.001	<0.001
eGFR (mL/min/1.73 m^2^)	103.9 (94.71,111.7)	97.24 (88.88,107.57)^**^	73.67 (56.94,102.43)^***,††^	<0.001	<0.001
Sclerostin (pmol/L)	28.33 (24.44,33.44)	25.07 (21.55,29.63)^**^	25 (21.58,29.97)^**^	<0.001	0.001
25 (OH)VD (nmol/L)	42.48 (30,57.23)	38.46 (26.56,51.53)	23.42 (12.7,36.6)^***,†††^	<0.001	<0.001
Use of metformin (Yes/No)	91/104	33/45	13/22	0.356	0.525
Use of statins (Yes/No)	30/165	16/62	6/29	0.31	0.593
Use of vitamin D supplements (Yes/No)	15/180	14/64	9/26	0.001	0.003

Data are presented as mean ± SD, standard deviation or median (interquartile range).

*P*_a_, p-value for the comparison between the T2DM group and DKD group.

*P*_b_, p-value for the overall comparison among the three groups (Normal albuminuria, Microalbuminuria, and Macroalbuminuria).

*Post-hoc* comparison: ^*^P < 0.05, ^**^P< 0.01, ^***^P < 0.001 for Microalbuminuria and Macroalbuminuric groups compared with the (Normal albuminuria group; ^†^*P* < 0.05, ^††^*P* < 0.01, ^†††^*P* < 0.001 for Macroalbuminuric group compared with the Microalbuminuria group.

BMI, body mass index; SBP, systolic blood pressure; DBP, diastolic blood pressure; FBG, fasting blood glucose; HbA1c, glycated hemoglobin; TC, cholesterol; TG, triglyceride; HDL, high-density lipoprotein; LDL, low-density lipoprotein; UA, uric acid; Scr: serum creatinine; eGFR, estimated glomerular filtration rate; 25 (OH)VD, 25-hydroxy vitamin D.

### Serum sclerostin and 25(OH)VD levels in T2DM patients with and without kidney disease

**Table 1**, **Figure 2** show that serum sclerostin and 25(OH)VD levels were decreased in the DKD group compared with the T2DM group. Further division of DKD patients into microalbuminuria and macroalbuminuria subgroups based on UACR revealed that serum sclerostin levels were significantly lower in both micro- and macroalbuminuria groups compared to the normal albuminuria group (*P<* 0.05), with no statistically significant difference between these two subgroups. Serum 25(OH)VD levels decreased with progression of albuminuria, being significantly lower in the macroalbuminuria subgroup than in both the normal albuminuria and microalbuminuria subgroups (P< 0.05), whereas no statistically significant difference existed between the normal and microalbuminuria subgroups.

**Figure 2:**
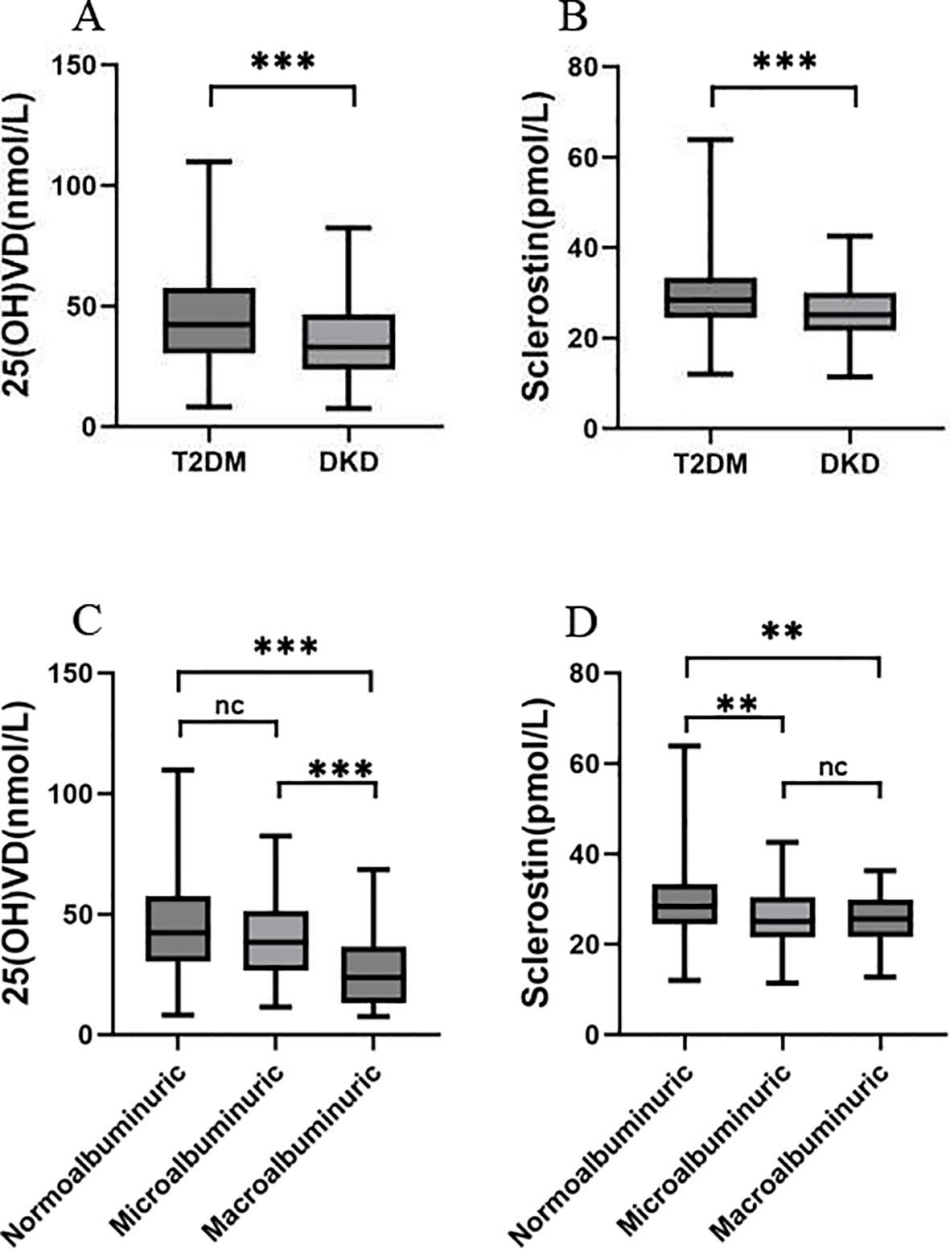
Box plots showing the distribution of serum sclerostin and 25(OH)VD levels across different patient groups. **(A, B)** show the differences between patients with T2DM and those with DKD. **(C, D)** present a stratified analysis of DKD patients based on albuminuria levels. Statistical significance is denoted as follows: ***P < 0.001, **P< 0.01, *P < 0.05. nc, no statistical significance.

### Relationship between UACR、serum sclerostin level and various parameters

The correlation analysis results showed (**Table 2**) that UACR was positively correlated with blood pressure, FPG, HbA1c, TG, UA, and Scr, and negatively correlated with eGFR, sclerostin, and 25(OH)VD (*P* < 0.05). Serum sclerostin levels showed a negative correlation with UACR and HbA1c, and a significant positive correlation with 25(OH)VD (r = 0.167, *P* = 0.003).

**Table 2:** Correlation analyses of UACR and serum sclerostin level with various parameters.

Variable	UACR (mg/g)		Sclerostin (pmol/L)	
	r	*P*	r	*P*
Age (years)	0.01	0.99	0.075	0.188
Duration (years)	0.099	0.084	0.053	0.358
Gender	-0.052	0.365	0.001	0.987
BMI (kg/m^2^)	0.77	0.178	0.002	0.975
SBP (mmHg)	0.261	<0.001	-0.02	0.729
DBP (mmHg)	0.142	0.013	-0.102	0.073
FPG (mmol/L)	0.137	0.016	0.01	0.861
HbA1c (%)	0.256	<0.001	-0.141	0.014
TC (mmol/L)	0.107	0.062	-0.058	0.31
TG (mmol/L)	0.136	0.018	0.021	0.714
HDL-C (mmol/L)	0.018	0.749	-0.009	0.871
LDL-C (mmol/L)	0.103	0.075	-0.046	0.428
UA (mmol/L)	0.166	0.004	-0.036	0.535
Scr (mg/dL)	0.167	0.004	-0.06	0.296
eGFR (mL/min/1.73 m^2^)	-0.175	0.002	0.04	0.948
UACR (mg/g)	—	—	-0.197	<0.001
Sclerostin (pmol/L)	-0.197	<0.001	—	—
25(OH)VD (nmol/L)	-0285	<0.001	0.167	0.003
Use of metformin (Yes/No)	-0.059	0.308	-0.088	0.126
Use of statins (Yes/No)	0.066	0.246	0.118	0.043
Use of vitamin D supplements (Yes/No)	0.127	0.026	-0.122	0.032

### Sclerostin and 25(OH)VD are independent factors for DKD

To examine the independent effects of serum sclerostin and 25(OH)VD on DKD, we constructed three stepwise adjusted binary logistic regression models (**Table 3**). Model 1 (without adjusted variables) demonstrated that both factors were significantly associated with DKD (*P* < 0.05). After stepwise adjustment for demographic characteristics and relevant clinical indicators (Models 2 and 3), the association between both factors and DKD remained statistically significant (*P* < 0.05). This robust finding indicates that serum sclerostin and 25(OH)VD are independent influencing factors for DKD.

**Table 3:** Binary logistic regression for the associations of sclerostin and 25 (OH)VD with DKD.

Variable	Model 1		Model 2		Model 3	
	OR (95%CI)	*P*-value	OR (95%CI)	*P*-value	OR (95%CI)	*P*-value
Sclerostin	0.944 (0.91,0.97)	0.002	0.942 (0.905,0.981)	0.004	0.942 (0.903,0.981)	0.004
25 (OH)VD	0.973 (0.959,0.987)	<0.001	0.972 (0.956,0.988)	<0.001	0.978 (0.961,0.995)	0.011

Model 1: without adjusted variable;

Model 2: adjusted for age, sex, duration, blood pressure and eGFR;

Model 3: model 2 with additional adjustment for fasting blood glucose and HbA1c.

### Predictive efficacy of serum sclerostin and 25-(OH)VD in predicting DKD

**Figure 3:**
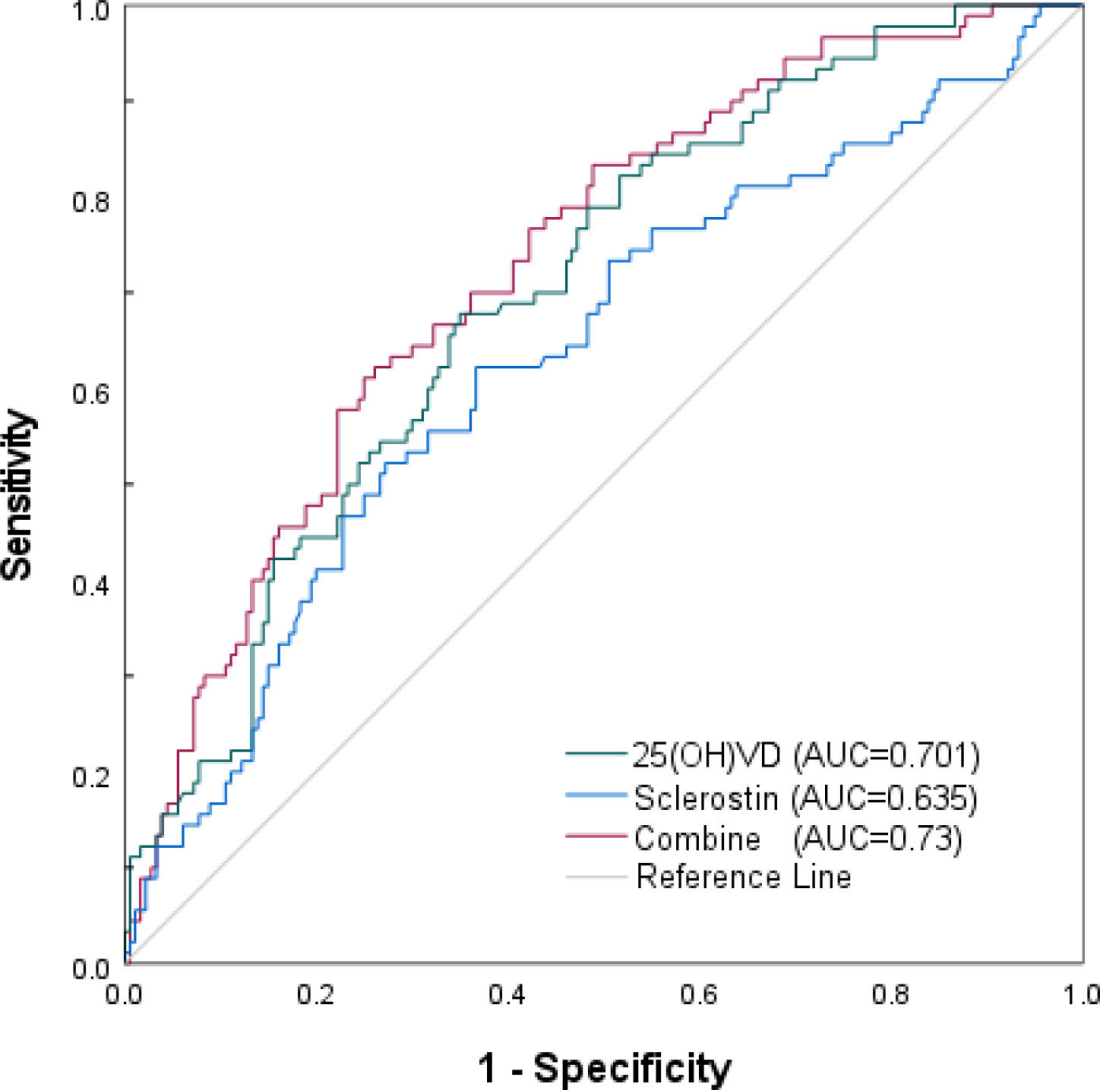
ROC analysis of serum sclerostin and 25(OH)VD for DKD.

To exclude the direct influence of oral vitamin D supplements on outcomes, we performed ROC curve analysis in patients not taking oral vitamin D to evaluate the diagnostic capability of serum sclerostin and 25(OH)VD, both individually and in combination, for DKD. The results (**Table 4**, **Figure 3**) showed that the area under the curve (AUC) for predicting T2DM with albuminuria was 0.635 for serum sclerostin alone and 0.701 for 25(OH)VD alone. The combined AUC for diagnosis was 0.73, with a sensitivity of 61.1% and specificity of 75%. .

**Table 4:** The predictive value Sclerostin and 25 (OH)D alone and in combination for DKD.

Indexes	AUC	95%CI	Cut-off	Sensitivity	Specificity
sclerostin	0.635	0.564~0.706	25.17	52.2%	72.8%
25(OH)VD	0.701	0.638~0.765	35.59	67.8%	65%
Combination	0.73	0.668~0.792		61.1%	75%

## Discussion

The prevalence of DKD continues to rise, with complex pathogenesis and subtle early clinical manifestations. It is necessary to identify early non-invasive biochemical markers for screening DKD. This study found that compared with T2DM alone, DKD patients exhibited decreased serum sclerostin and 25(OH)VD levels. Both are independent factors for DKD, and their combined diagnosis holds clinical value, offering novel indicators for early clinical diagnosis.

25(OH)VD participates in multiple cellular functions and has been found to be commonly deficient in patients with DKD (24, 25). Our findings are consistent with these previous conclusions. Sclerostin is considered a novel endocrine factor secreted by osteocytes, and studies indicate it plays a crucial role in glucose and lipid metabolism. Serum sclerostin levels are significantly increased in diabetic and obese populations (26–28). This study found decreased serum sclerostin levels in the DKD group compared to T2DM patients with normal proteinuria. This suggests that when T2DM progresses to DKD, sclerostin levels decrease despite being elevated by metabolic dysregulation. It has been demonstrated in basic research that sclerostin gene deletion promotes renal interstitial fibrosis. Sclerostin levels are significantly decreased in the renal tubules of patients with chronic kidney disease and in mouse models, while sclerostin treatment or overexpression can effectively alleviate renal fibrosis, demonstrating a protective role for sclerostin in kidney disease (18). Correlation analysis in this study revealed significant negative correlations between sclerostin, 25(OH)VD, and UACR. Even after adjusting for multiple confounding factors in the logistic regression model, sclerostin and 25(OH)VD remained independent protective factors for DKD. Therefore, sclerostin holds potential value for early screening of DKD patients.

It is worth noting that the role of sclerostin in DKD remains controversial (16). Studies have shown that circulating sclerostin is closely associated with vascular calcification. Some research supports its potential to alleviate vascular calcification and improve patient survival rates (29, 30). Conversely, other researchers indicate that elevated levels of sclerostin may induce vascular calcification, increase mortality, and show a positive correlation with the stages of CKD (31, 32). This may be closely related to the dual role of the Wnt/β-catenin signaling pathway in DKD. Moderate activation of this pathway is crucial for renal repair and regeneration, whereas its prolonged excessive activation induces renal fibrosis. As a natural antagonist of Wnt/β-catenin signaling pathway, changes in sclerostin levels exert differential effects on this pathway. Furthermore, the low-density lipoprotein receptor 5 (LRP5), a key cell surface receptor initiating Wnt signaling, is abundant and functionally significant in renal tubules (33, 34). In models of type 2 diabetes and chronic kidney disease, LRP5 upregulation promotes fibrosis, whereas LRP5 knockout suppresses TGF-β/SMAD signaling to mitigate renal interstitial fibrosis and injury (33). We hypothesize that during DKD progression, decreased circulating and local sclerostin expression weakens inhibition of Wnt/β-catenin signaling, leading to its prolonged overactivation and subsequent induction of renal fibrosis. Simultaneously, the relatively elevated expression of LRP5 in the renal tubules of patients with diabetes and kidney disease further promotes renal injury. Additionally, variations in study populations, baseline disease states, relevant drug treatments, and experimental protocols contribute to divergent outcomes. This study found that 25(OH)VD levels showed a decreasing trend in the microalbuminuria group compared to the normal albuminuria group, though the difference did not reach statistical significance, which may be related to vitamin D supplementation in some patients. Additionally, no statistically significant difference in sclerostin levels was observed between the microalbuminuria and macroalbuminuria groups, suggesting that larger clinical samples may be required for further validation.

There is a close correlation between sclerostin and 25(OH)VD. Previous studies have demonstrated significantly reduced sclerostin levels in newborns of vitamin D-deficient mothers, while serum sclerostin levels were markedly higher in vitamin D-supplemented patients compared to non-supplemented individuals (35, 36). Basic experiments confirmed that supplementation with vitamin D analogues can block Wnt/β-catenin signaling, thereby improving proteinuria and renal injury (37). This study revealed a significant positive correlation between sclerostin and 25(OH)VD levels. Furthermore, after excluding the influence of exogenous vitamin D supplementation, ROC curve analysis demonstrated that the combined diagnostic value of sclerostin and 25(OH)VD for T2DM with albuminuria surpassed that of either marker alone. These findings suggest a potential synergistic and intrinsically linked role for both factors in DKD, though the specific mechanism requires further investigation.

This study indicates that bone metabolism-related factors (sclerostin and 25(OH)VD) exert protective effects in DKD. Both factors may play important roles in the anti-fibrotic process by inhibiting the Wnt/β-catenin signaling pathway. In addition, 25(OH)VD may also contribute to renal protection through its anti-inflammatory and metabolic regulatory functions (38, 39). Numerous studies have confirmed the critical role of anti-inflammatory, anti-fibrotic, and metabolic regulatory effects in the treatment of DKD (5, 8, 40, 41). Therefore, future research should further elucidate the specific mechanisms of sclerostin and 25(OH)VD in DKD through basic experiments, providing new insights for the early diagnosis and intervention of DKD as well as for a deeper understanding of the regulatory network of the “bone-kidney axis”.

## Limitations

This study has several limitations. First, it is a single-center, cross-sectional study with a relatively small sample size, which limits the generalizability of the findings. Second, the cross-sectional design only allows for correlation analysis and cannot establish causality or capture dynamic changes in the biomarkers over time. Although we observed a correlation between serum sclerostin and 25(OH)VD, the exact relationship between these two markers still needs to be clarified in future prospective studies. Third, the lack of an independent external validation cohort means that the clinical utility of the combined diagnostic model should be confirmed in larger, more diverse populations. Finally, although we adjusted for multiple known confounders, unmeasured factors may still have influenced the results.

## Conclusion

This study confirms significantly reduced serum sclerostin and 25(OH)VD levels in DKD patients, with both markers acting as independent protective factors. Their combined assessment demonstrates promising predictive value for early identification of DKD and offers clinical insight into the interaction between bone metabolism and kidney pathology.

## Data Availability

The original contributions presented in the study are included in the article/supplementary material. Further inquiries can be directed to the corresponding authors.
